# Gamma knife radiosurgery for movement disorders: a concise review of the literature

**DOI:** 10.1186/1477-7819-8-61

**Published:** 2010-07-21

**Authors:** Ameer L Elaimy, Benjamin J Arthurs, Wayne T Lamoreaux, John J Demakas, Alexander R Mackay, Robert K Fairbanks, David R Greeley, Barton S Cooke, Christopher M Lee

**Affiliations:** 1 Gamma Knife of Spokane, 910 W 5th Ave, Suite 102, Spokane, WA 99204, USA; 2 University of Washington School of Medicine, 325 9th Ave, Seattle, WA 98104, USA; 3 Spokane Brain & Spine, 801 W 5th Ave, Suite 210, Spokane, WA 99204, USA; 4 Cancer Care Northwest, 910 W 5th Ave, Suite 102, Spokane, WA 99204, USA; 5 MacKay & Meyer MDs, 711 S Cowley St, Suite 210, Spokane, WA 99202,USA; 6 Northwest Neurological PLLC, 507 S Washington St, Suite 101, Spokane, WA 99204, USA

## Abstract

Medication is the predominant method for the management of patients with movement disorders. However, there is a fraction of patients who experience limited relief from pharmaceuticals or experience bothersome side-effects of the drugs. Deep brain stimulation (DBS) and surgical lesioning of the thalamus and basal ganglia are respected neurosurgical procedures, with valued success rates and a very low incidence of complications. Despite these positive outcomes, DBS and surgical lesioning procedures are contraindicated for some patients. Stereotactic radiosurgery with the Gamma Knife (GK) has been used as a lesioning technique for patients seeking a non-invasive treatment alternative and for medication-intolerable patients, who are unable to undergo DBS or lesioning due to comorbid medical conditions. Tremors of various etiologies are treated using GK thalamotomy, which targets the ventralis intermedius nucleus. GK thalamotomy produces favorable outcomes when treating tremors, with success rates ranging from 80-100%. In contrast, GK pallidotomy targets the internal globus pallidus, and is used in treating bradykinesia, rigidity, and dyskinesia. Although radiosurgery has proven beneficial for tremors, radiosurgical pallidotomy for bradykinesia, rigidity, and dyskinesia remains questionable, with mixed success rates in the literature that ranges from 0-87%. We suggest that GK thalamotomy be offered along with other neurosurgical approaches as a feasible treatment option to patients who prefer the non-invasive nature of radiosurgery and to those who are unqualified candidates for the neurosurgical alternatives. Also, we advise that patients with bradykinesia, rigidity, and dyskinesia be educated about the variability in the literature pertaining to GK pallidotomy before proceeding with treatment.

## Background

Pharmacotherapy is the general treatment method for patients who suffer from movement disorders. Even though a large proportion of patients are able to manage their condition with medication, there is still a small amount of patients who do not experience significant relief from pharmaceuticals, thus, seek out other treatment modalities. Deep brain stimulation (DBS) and surgical lesioning of the thalamus and basal ganglia are respected and well-studied neurosurgical procedures that come with a low incidence of potential side-effects. However, there is a subset of patients with movement disorders who are not qualified candidates for invasive neurosurgery. This population of patients consists of those who use anticoagulants, those who have advanced cardiac or respiratory disease, those who are known to be noncompliant, those who are of advanced age, and those who elect to not proceed with neurosurgery. Despite the fact that radiofrequency (RF) neurosurgical lesioning has shown success in many patients, there is still a possibility for patients to encounter a wide array of side-effects. These include intracerebral or extracerebral hemorrhage, seizures, infection, brain displacement, tension pnemocephalus, and direct injury from probe placement [1]. Stereotactic radiosurgery using the gamma knife (GK) is a non-invasive alternative modality for lesioning intracranial structures.

The first cobalt-60-based GK device dates back to Sweden in 1968, where Professor Lars Leksell's intention was to create precisely located, well-circumscribed lesions in the brain in a minimally-invasive fashion [2]. Between 1968 and 1982, a total of 762 patients underwent treatment with the cobalt-based GK unit. Only 5 of the 762 patients were treated for Parkinsonism, but this historic study shows that the idea of treating movement disorders using radiosurgical techniques is not a recent advance and has evolved considerably over the past four decades.

In recent years, thalamotomy and pallidotomy with the GK have been used to treat a variety of movement disorders. Specifically, GK thalamotomy targets the ventralis intermedius nucleus (VIM) of the thalamus, and is used in treating essential, Parkinsonian, and other types of tremors. Evidence suggests that GK thalamotomy produces favorable results when treating tremors, offering a safe alternative to RF thalamotomy and DBS [1,3-9]. GK pallidotomy targets the internal globus pallidus (Gpi) of the basal ganglia, and is used in treating bradykinesia, rigidity, and dyskinesia. However, a variety of outcomes have been reported when using radiosurgical pallidotomy, thus, it remains a controversial procedure [1,3,10,11].

We present a brief modern review of published data on the effectiveness of GK thalamotomy and pallidotomy in the treatment of patients with movement disorders.

### Review

*GK thalamotomy for tremor treatment *(See Table 1 for data summary).

**Table 1:** GK thalamotomy for tremor treatment

1^st ^Author (Year)	# Patients (Age Range)	Radiation Dose (Gy)	Follow-up (Range)	Improvement Rate	Complications Observed	Complication Rate
Young [9] (2010)	161 (18-93 yrs)	141-152	Mean: 44 ± 33 months	Drawing: 81% Writing: 77%	sensory loss, motor impairments, dysarthria	8.4%
Lim [12] (2010)	18 (64-83 yrs)	130-140	Mean: 19.2 months (7-30 months)	NR	edema, hemorrhage, dysarthria, hemiparesis, lip and finger numbness	16.7%
Kondziolka [5] (2008)	31 (52-92 yrs)	130-140	Median: 36 months (4-96 months)	92%	hemiparesis, dysphagia, dysarthria	7.7%
Duma [3] (1999)	38 (60-84 yrs)	120-160	Median: 30 months (6-72 months)	90%	dysarthria	2.6%
Young [1] (1998)	27 (73.3 ± 7.2 yrs**)	120-160	Mean: 22.3* months (12-44* months)	89%	none	0%
Young [8] (2000)	PD: 102 (71.3 ± 8 yrs) ET: 52 (73.8 ± 9.4 yrs) Other: 4 (64.3 ± 7 yrs)	120-160	<12-96 months	PD: 88.3% ET: 92.1% Other: 50%	balance disturbance, paresthesias, weakness, dysphasia	1.3%
Niranjan [7] (1999)	12 (38-78 yrs)	130-150	Median: 24 months (4-40 months)	100%	dysarthria, weakness	8.3%
Niranjan [15] (2000)	11 (38-92 yrs)	130-150	Median: 6 months (2-11 months)	100%	dysarthria, weakness	9.1%
Mathieu [6] (2007)	6 (31-57 yrs)	130-150	Median: 27.5 months (5-46 months)	100%	hemiparesis	16.7%
Friedman [4] (1999)	15 (37-84 yrs)	120-140	3 months	93.3%	edema, incoordination, action tremor	46.7%

ET = essential tremor; NR = not reported; PD = Parkinson's disease

*Data includes pallidotomy patients

**Data includes only patients assessed by an independent team

In 2010, Young *et al*. [9] published a study analyzing 161 patients who were treated for ET with GK thalamotomy. The main clinical scale utilized to assess tremor control was the Fahn-Tolosa clinical rating scale. The authors reported statistically significant (P < 0.0001) differences in both drawing scores (81% of patients showed improvements) and writing scores (77% of patients showed improvements), with a mean follow-up of 44 ± 33 months. Overall, 14 (8.4%) patients suffered from post-operative complications, which included limited sensory loss contralateral to the side of the procedure, motor impairments, and difficulties with speech. In the same year, Lim *et al*. [12] investigated the role of GK thalamotomy in 18 patients with disabling tremor from either ET or Parkinson's disease (PD). The authors utilized the clinical Fahn-Tolosa scale and the United Parkinson's Disease Rating Scale (UPDRS) to assess potential tremor improvements. Follow-up ranged from 7 to 30 months (mean of 19.2 months). It was reported that patients significantly improved (P = 0.03) in activities of daily living scores. However, 3 (16.7%) patients encountered toxicities from the procedure. The observed complications from radiosurgery included edema, hemorrhage, dysarthria, hemiparesis, and lip and finger numbness.

In 2008, Kondziolka *et al*. [5] performed a study where 31 patients with ET were treated with thalamotomy using GK radiosurgery. All patients were considered unqualified candidates for neurosurgery. The Fahn-Tolosa tremor scale was used to provide an objective measurement of response to treatment. The authors reported statistically significant improvements in both the mean tremor score (P < 0.000015) and mean handwriting score (P < 0.0002) following radiosurgery (median follow-up of 36 months). Of the evaluated patients, 18 (69%) exhibited improvements in their action tremor and handwriting scores, 6 (23%) exhibited improvements in only their action tremor, and 3 (12%) did not exhibit compelling improvements in either variable. One patient suffered from transient mild right hemiparesis and dysphagia, while a separate patient also developed mild right hemiparesis and difficulties in their speech following radiosurgery.

An initial review on movement disorders was performed in the past by Duma *et al. *[3]. Over a seven year period, 38 patients with disabling tremor from PD underwent thalamotomy using the GK. Patients were assessed using the UPDRS. 42 thalamic lesions were created in these 38 patients, and 90% were deemed successful, with respect to tremor control. Young *et al. *[1] also performed a study that evaluated the safety and efficacy of GK thalamotomy in the treatment of tremors. The UPDRS and Hoehn and Yahr ratings determined by trained specialists were utilized. Overall, an 88.9% success rate was reported in their 27 patients suffering multiple types of tremors. More specifically, 16 patients were treated for Parkinsonian tremor, 8 were treated for ET, 2 were treated for tremor following cerebral infarctions, and 1 patient was treated for a tremor following a bout of encephalitis. After a mean follow-up of 22.2 months, 19 patients experienced complete or nearly complete resolution of tremor and 5 patients were nearly tremor free. Young *et al. *[8] also completed an additional study investigating the long-term effects of GK thalamotomy for disabling tremor and obtained favorable results. Patients were evaluated by blind evaluations, the UPDRS, and the Fahn-Tolosa tremor scale. After a mean follow-up of 52.5 months, 88.3% of PD patients became fully or nearly tremor free. At 12 months post-operation, 92.1% of ET patients were fully or nearly tremor free. 88.2% of these ET patients maintained excellent tremor control 48 months or more following radiosurgery. Only 50% of patients with other forms of tremor experienced notable improvements.

To compare the surgical approaches for the management of tremor, Niranjan *et al. *[7] analyzed the outcomes of patients treated with GK thalamotomy, RF thalamotomy, and DBS. Out of the 13 patients that underwent RF thalamotomy, 5 (39%) had complete arrest of tremor, 6 (46%) had a significant reduction, and 2 (15%) had approximately 50% tremor reduction. All 11 DBS patients experienced excellent tremor control immediately after surgery, and it was reported that only 2 (18.2%) of those patients' tremor reoccurred. 10 (83.3%) noted excellent tremor relief and 2 (16.7%) experienced good relief. Niranjan's results with GK thalamotomy correlates with studies done by Jankovic *et al. *[13] and Fox *et al. *[14] with RF thalamotomy. Their reported success rates were 90% and 91%, respectively.

GK thalamotomy is also a worthy treatment option to consider for patients who struggle with tremors caused by Multiple Sclerosis (MS). Mathieu *et al. *[6] created radiosurgical thalamic lesions in six patients with MS-related tremors and recorded advantageous results. Patient results were assessed using the Fahn-Tolosa tremor scale. After a median follow-up time of 27.5 months, it was documented that radiosurgery was beneficial to every patient. Niranjan *et al. *[15] explored the role of GK thalamotomy in the management of ET and MS-related tremor in 12 patients of advanced age (median of 75 years). Patient outcomes were also assessed using the tremor scale diagrammed by Fahn-Tolosa. Of the 11 evaluable patients, 9 (81.8%) reported excellent tremor control and 2 (18.2%) announced a satisfactory improvement with their tremor.

Two issues pose potential challenges with radiosurgical thalamotomy: the time interval between treatment and effect and the variability of the thalamic reaction, and inability to predict the potential subsequent side-effects for specific patients. Both of these issues were demonstrated in a study done to evaluate the survival of neurons adjacent to the thalamic lesion after GK thalamotomy by Ohye *et al. *[16]. They performed a total of 36 thalamotomies in 31 patients and analyzed the treatment outcomes. It was noted in this analysis that in the majority of patients, tremor reduction started approximately one year after irradiation. The delay in treatment effect may not be desired by some patients. Based on MRI data, two types of tissue reactions were observed: a simple oval lesion and one of a complex irregular shape. There was no correlation between the tissue reaction and tremor outcome. Unlike DBS and stereotactic lesioning, where subsequent side-effects can be predicted by neurological physiological responses during the procedure, there are no predictors prior to GK radiosurgery for the type of resultant lesion observed. In some patients, the lesion may extend into the internal capsule or medial thalamic region, causing a variety of delayed-onset complications months after GK radiosurgery that cannot be anticipated [16].

*GK pallidotomy for bradykinesia, rigidity, and dyskinesia treatment *(See Table 2 for data summary).

**Table 2:** GK pallidotomy for bradykinesia, rigidity, and dyskinesia treatment

1^st ^Author (Year)	# Patients (Age Range)	Radiation Dose (Gy)	Follow-up (Range)	Improvement Rate	Complications Observed	Complication Rate
Duma [3] (1999)	18 (59-85 yrs)	120-160	Median: 8 months (6-40 months)	33.3%	homonymous visual field cut, dysphagia, dysarthria, hemiparesis, hemianesthesia, worse gait	50%
Friedman [10] (1996)	4 (61-74 yrs)	180	18 month interval	0%	dementia, psychosis	25%
Young [1] (1998)	28 (68.2 ± 10.2 yrs**)	120-160	Mean: 22.3* months (12-44* months)	Bradykinesia/Rigidity rate: 64.3% Dyskinesia rate: 85.7%	homonymous hemianopsia	3.6%
Young [11] (1998)	29	120-140	Mean: 20.6 months (6-48 months)	Bradykinesia/Rigidity rate: 65.5% Dyskinesia rate: 86.6%	homonymous hemianopsia	3.4%

*Data includes thalamotomy patients

**Data includes only patients assessed by an independent team

In contrast to radiosurgical thalamotomy, controversy exists regarding the effectiveness of GK pallidotomy for bradykinesia, rigidity, and dyskinesia. Duma *et al. *[3] performed a study investigating outcomes of GK pallidotomy. In contrast to the prior section on thalamotomy, they reported a lack of faith in the procedure which targets the basal ganglia. Similar to the prior section on GK thalamotomy, the authors used the UPDRS to assess patient outcomes. A total of 18 patients underwent stereotactic lesioning in the basal ganglia for bradykinesia, rigidity, and dyskinesia related to PD. Only 6 (33%) patients showed transient improvement in rigidity and dyskinesia. Three (17%) patients displayed no changes, and 9 (50%) were worsened by the treatment. The complications from treatment included homonymous visual field cuts, dysphagia, dysasthria, hemianesthesia, hemiparesis, and a worse gait. As with GK thalamotomy, the size of the lesions, thus observed complications, created by GK pallidotomy cannot be anticipated. Also, to explain the high complication rate, the authors hypothesized that lesion creation in the basal ganglia is more difficult than in the thalamus due to a greater likelihood of perforating arteries [3] In addition to arterial infarction, the authors hypothesized that there is a differential sensitivity to radiation between the VIM and Gpi. This is because the pallidum has previously been known to show an increased sensitivity to hypoxia [3]. Also, because the iron concentration in the pallidum tends to increase with age, it has been thought that the excess iron catalyzes undesirable reactions, thus, leading to the formation of free radicals [3]. Friedman *et al. *[10] witnessed outcomes with GK pallidotomy comparable to that of Duma *et al. *[3] Only four patients participated in the study, and none of them exhibited compelling improvements. Complications were seen in one patient, who became psychotic and demented following radiosurgery.

Conversely, additional research studies from single institutions have reported positive outcomes in radiosurgical pallidotomy. Young *et al. *[11] performed a study comparing the outcomes of RF pallidotomy and GK pallidotomy for patients with PD. The UPDRS and Hoehn and Yahr ratings were utilized. In 29 patients, the pallidotomies were performed radiosurgically, and 22 patients had the open-skull RF method performed. Before surgery, 15 of the 29 radiosurgery patients experienced dyskinesias, and 13 (86.6%) had complete or nearly complete relief of that symptom postoperatively. Out of the 22 RF patients, 12 experienced dyskinesia preoperatively, and 10 (83.3%) of those patients had complete or nearly complete relief after surgery. Bradykinesia and rigidity were present in every patient preoperatively, and 19 (65.5%) of the GK patients and 14 (63.6%) of the RF patients had significant improvement in those symptoms. One patient in the GK group developed a homonymous hemianopsia nine months after treatment and five RF patients became transiently confused after surgery.

## Conclusions

The goal of this report is to provide a concise review of the literature on the efficacy and potential side-effects of GK radiosurgery in the treatment of patients with movement disorders. As seen in the reported research, thalamotomy with the GK is an effective and non-invasive alternative in treating tremors, with success rates ranging from 80-100%. Additionally, because of the non-invasive lesioning technique associated with radiosurgical thalamotomy, the procedure comes with a different risk profile than the open-skull neurosurgical methods. On the contrary, GK pallidotomy has shown mixed outcomes in the treatment of bradykinesia, rigidity, and dyskinesia. The inconsistency of radiosurgical pallidotomy is demonstrated in the available literature, with success rates that range from 0-87%. However, studies on radiosurgical pallidotomy have not been as extensive as those on radiosurgical thalamotomy, so less is known about the procedure and there remains room for continued research and improvements. We suggest that GK thalamotomy should be mentioned as a viable treatment option to tremor patients who prefer the non-invasive aspects of radiosurgery and to the fraction of medication-intolerable patients, who are ineligible to undergo RF thalamotomy or DBS. We also recommend that patients should be educated about the variability in the literature pertaining to GK pallidotomy and need for further study before proceeding with radiosurgery treatment.

## Competing interests

The authors declare that they have no competing interests.

## Authors' contributions

ALE and CML reviewed relevant literature for this review and drafted the manuscript. BJA, WTL, JJD, ARM, RFK, DRG, and BSC provided expertise relevant to this review and helped draft the manuscript. All authors read and approved the final manuscript.
